# Thinking Aloud About Poverty and Health in Rural Mississippi

**Published:** 2007-06-15

**Authors:** Leonard Jack

**Affiliations:** School of Health Sciences and Professor of Social Work, School of Social Work, Jackson State University

Poverty and health status are interrelated, and their effects on each other are often bidirectional: poverty leads to poor health and poor health leads to poverty (1,2). In addition, life challenges associated with poverty, whether short- or long-term, create conditions that reduce household savings, lower learning ability, and reduce physical and emotional well-being (1), all of which endanger people's health (1,2). Many Mississippians, especially the 51% who live in rural counties (3), experience poverty levels that are hard to imagine for most Americans. In particular, in recent years poor Mississippians faced heavy job losses in industries that once provided high wages and good benefits (4). These job losses led to decreases in annual income, increases in bankruptcies, and a declining number of people with health insurance (5). For example, from 2000 to 2003, median household incomes fell by $3,910 to $32,728, and the number of poor people increased by 38,000 to 456,000 (5). Regardless of location, poor people are more likely than affluent people to lack health insurance (6), so we can assume that many of the 19% of respondents to the Mississippi Behavioral Risk Factor Surveillance System who said they had no health insurance are poor (7).

Mississippi is one of the poorest states in the nation (5). More than the poor in other states, the poor in Mississippi receive inadequate education, have limited access to quality health care, and experience personal and environmental risks that lead to poor nutrition (5). Understanding the interaction between poverty and health in Mississippi requires a candid discussion about poverty in the United States as a whole. Those involved in this discussion should be public health professionals, clinicians, policy makers, and professionals from fields (e.g., labor, agriculture) not always associated with solving health-related issues. This discussion must be public and include topics such as the root causes of poverty; the physical and emotional health problems common to poor people, regardless of geographic location; the characteristics of poor people; the personal, family, community, state, and national resources needed to prevent poverty and its related adverse health effects; and the ingrained perceptions that many middle-income and affluent people have about the poor. Without frank discussions on those topics, devising innovative solutions to poverty-related health problems in Mississippi (or the United States as a whole) will be difficult.

Poverty in Mississippi is similar to or worse than the poverty in some third world countries (8), and articles in Mississippi newspapers frequently report on people's experiences with poverty (8). Because Mississippi ranks high among states with a disproportionate burden of chronic diseases, teenage pregnancy, and infant mortality, national newspapers also cover poverty in Mississippi. A July 2004 *Washington Post* article entitled "Poverty Tightens Grip On Mississippi Delta: Number of Young Rural Poor Rises, Study Says'' reported that 55% of households in Coahoma, Mississippi, a rural community of 350, had incomes of less than $15,000 per year, well below the federal poverty line of $18,850 for a family of four (9). The newspaper article continued:

The human faces of poverty for many Americans are the inner-city homeless who sleep on grates, beg on corners and line up, mornings and afternoons, at local parks for a cup of soup and a sandwich. But of the 50 counties with the highest child-poverty rates, 48 are rural American. Compared with urban areas, unemployment is typically higher, education poorer and services severely limited because people are so spread out . . .. A lot of people believe it's got to be cheap to live there [rural area] and food has got to be more available. But cheap is relative to income. Your ability to move yourself around is limited. There is no public transportation.

And the effects of poverty go beyond the individual. Poverty affects a community's ability to support capital improvements; to build and maintain schools; to provide health care services; and to provide policing, social, and sanitation services. It seems obvious that poor people experience a high burden of disease, often die prematurely, and have a poor quality of life (10). What is not so obvious is that the health of the poor appears to worsen as the national gap between rich and poor widens (10).

George Kaplan and colleagues defined measures of income inequality and compared them with various rates of disease and social problems (e.g., incarceration, unemployment) in each of the 50 U.S. states (10). They found that the greater the inequality in the distribution of income, the higher the rates of 1) unemployment and incarceration, 2) people receiving income assistance and food stamps, and 3) people without medical insurance. States with the greatest reported inequality of income distribution spent less per person on education, had fewer books per person in schools, and had poorer education performance (e.g., poorer reading skills and math scores, lower rates of high school completion) than states where the gap between rich and poor is not as pronounced (10). The researchers also reported that states with the greatest inequality of income had the highest costs per person for police protection and medical care (10).

Regardless of people's race, short-term poverty can have as much of a negative effect on their health as long-term poverty. Using data from 1968 through 1995 from the American Panel Study of Income Dynamics, McDonough and colleagues (11) found that people who were never poor were the healthiest and people who were always poor were the least healthy. Surprisingly, they also found that people who overcame poverty or became poor over time — especially if they were elderly, not well educated, and not white — had a similar health risk to the risk of those who were always poor.

Opinions on how to overcome poverty in Mississippi, the nation, and around the world range from putting the onus on individuals to emerge out of poverty by their own efforts to requiring increases in government spending on antipoverty programs through increased taxes (12). Some believe that tax cuts promote economic growth, which then improves economic equality for all. Others believe a combination of individual responsibility and government programs is required (12). One factor is certain: poverty challenges the belief that individuals are solely responsible for their own well-being. Without outside help, few among the poor can overcome limited access to good quality education for their children, limited means to purchase nutritional foods, and limited access to good quality health care.

The cycle of poverty and poor health requires a balance of interventions from public health professionals, environmentalists, and people working in areas that greatly affect health (e.g., labor, trade, agriculture). We must focus on the health consequences of poverty. By doing so, we can break the cycle of poverty leading to ill health and ill health leading to poverty. And we must focus not only on issues related to physical health, but also on issues related to mental health (e.g., isolation, hopelessness, chronic stress, depression) (13). Poor Mississippians, like most Americans who live in poverty, want desirable jobs so they can provide for their families, afford decent housing in safe neighborhoods, have their children attend and graduate from good schools, have access to good medical care, and be treated with respect despite their poverty.

International experts say that until poverty is reduced, health issues among the world's poor will look no different in the future than they do today (14). Acknowledging the effect of poverty on health is a start, but the real work involves public health professionals strategizing and working with traditional and nontraditional partners to reduce poverty. I am encouraged by the work of the World Health Organization (WHO), which recommends four strategies to improve the health of the poor throughout the world (15). These strategies are also relevant to improving the health of poor rural Mississippians:

- **Act on the determinants of health by influencing policy.** According to WHO, equitable distribution of the benefits of economic growth is central to reducing poverty. Maximize the health benefits of economic growth through public policies related to labor, trade, agriculture, environment, and health. Such policies affect people at each stage of life. Getting such policies implemented, however, requires collaborations and networks between public health and many other sectors of society.

- **Ensure that health systems serve the poor effectively.** Beyond ensuring that communities have the capacity to provide optimal health services, public health agencies must address the characteristics that cause health care systems to fail the poor. WHO recommends, at a minimum, that health care systems ensure access irrespective of income and that the poor are treated with dignity and respect, thus protecting the poor from unsafe practices and financial exploitation.

- **Focus on the health problems that disproportionately affect the poor.** WHO proposes providing governments with the tools and guidelines they need to set up the best and most cost-effective interventions to tackle health challenges that disproportionately affect the poor in their countries. Similarly, U.S. public health agencies need to provide Mississippi with technical assistance and resources so that its state and local health departments, other state agencies, universities, and nongovernmental organizations can set up interventions to prevent or control diseases that disproportionately affect poor rural Mississippians.

- **Reduce health risks through a broad approach to public health.** Improve poor people's access to basic public services (e.g., clean water, modern sanitation). In addition, recognize that poor people are more likely to be exposed to violence and environmental hazards and more likely to suffer as a result of conflicts and natural disasters than are affluent people. Planning and preparing for emergencies is particularly critical and requires participation not only by people with experience and expertise in first response and emergency management, but also by people from diverse groups (e.g., sanitation specialists, chronic disease specialists).

The consequences of poverty become abundantly apparent during natural disasters such as Hurricane Katrina. It is true that a natural disaster of Katrina's magnitude does not distinguish between rich and poor. However, as Milio (16) reported in 2006, "it is undeniable and troubling that the majority of those affected by Katrina were among our nation's poorest individuals and families even before the storm hit." Historically, public health specialized in responding to health crises during natural disasters by capturing and analyzing epidemiologic data and intervening to reduce or minimize the negative health consequences associated with the disaster itself. Recently, public health expanded its response to disasters by intervening to prevent the chronic diseases of those displaced by disasters from worsening. This change in policy is a good step toward helping poor people suffer less as a result of natural disasters.

But more needs to be done. Undeniably, the relationship between poverty and health is complex. Finding new methods of intervening will require many of us working in public health to think differently, partner differently, challenge stereotypes about the poor (17), and listen more carefully than ever to poor people themselves. Furthermore, public health professionals should consider incorporating successful clinical interventions into public health practice, collaborating with nontraditional partners (e.g., labor unions, public policy makers, trade associations), and researching the effectiveness of interventions in poor, underserved rural communities. In a speech delivered in 1964, Dr George James, Health Commissioner of the New York City Department of Health, commented (18):

Medicine is only part of the attack upon poverty. Just as we are learning in medicine to consider the whole human being and his entire family, so we are going to have to mount a comprehensive attack against poverty. Public health people alone cannot do it. Politicians alone cannot do it; nor can it be done just in the city, just in the country, or just in Appalachia or any other region. It must be across the board.

Recent public health research (19) explored the role of social determinants — largely how to measure their effects — in poverty, thus no longer ignoring their once minimized or dismissed role in shaping people's health. One caveat, however: even when public health has new ideas about how to improve poor people's health, we must be careful not to impose these new ideas on poor communities without consulting with its members first. Clearly, poor communities — indeed all communities, rich or poor — are more likely to accept and participate in public health interventions if they are developed in concert with community members and if they incorporate community competencies and assets than if the interventions are developed by outsiders without consultation with those who are supposed to benefit. However, making culturally tailored public health interventions available and providing access to health services is not sufficient if the underlying social determinants of poor health go ignored (19,20).

I invite my fellow public health professionals to join the fight against poverty and poverty-related ill health throughout the United States and the world. And I suggest that a good place to start a major offensive in that fight is rural Mississippi.
